# Mechanistic insights gained from cell and molecular analysis of the neuroprotective potential of bioactive natural compounds in an immortalized hippocampal cell line

**DOI:** 10.1371/journal.pone.0267682

**Published:** 2022-06-03

**Authors:** Harris A. Weisz, Deborah R. Boone, William S. Coggins, Gabrielle A. Edwards, Hannah E. Willey, Steven G. Widen, Dionicio Siegel, Andrew T. Nelson, Donald S. Prough, Helen L. Hellmich

**Affiliations:** 1 Department of Anesthesiology, The University of Texas Medical Branch at Galveston, Galveston, Texas, United States of America; 2 Department of Neurosurgery, The University of Arkansas for Medical Sciences, Little Rock, Arkansas, United States of America; 3 Tulane University School of Medicine, New Orleans, Louisiana, United States of America; 4 Department of Biochemistry & Molecular Biology, The University of Texas Medical Branch at Galveston, Galveston, Texas, United States of America; 5 Skaggs School of Pharmacy and Pharmaceutical Sciences, The University of California San Diego, San Diego, California, United States of America; 6 Department of Pathology, The University of Texas Southwestern Medical Center, Dallas, Texas, United States of America; Bangladesh Agricultural University, BANGLADESH

## Abstract

Evaluating novel compounds for neuroprotective effects in animal models of traumatic brain injury (TBI) is a protracted, labor-intensive and costly effort. However, the present lack of effective treatment options for TBI, despite decades of research, shows the critical need for alternative methods for screening new drug candidates with neuroprotective properties. Because natural products have been a leading source of new therapeutic agents for human diseases, we used an *in vitro* model of stretch injury to rapidly assess pro-survival effects of three bioactive compounds, two isolated from natural products (clovanemagnolol [CM], vinaxanthone [VX]) and the third, a dietary compound (pterostilbene [PT]) found in blueberries. The stretch injury experiments were not used to validate drug efficacy in a comprehensive manner but used primarily, as proof-of-principle, to demonstrate that the neuroprotective potential of each bioactive agent can be quickly assessed in an immortalized hippocampal cell line in lieu of comprehensive testing in animal models of TBI. To gain mechanistic insights into potential molecular mechanisms of neuroprotective effects, we performed a pathway-specific PCR array analysis of the effects of CM on the rat hippocampus and microRNA sequencing analysis of the effects of VX and PT on cultured hippocampal progenitor neurons. We show that the neuroprotective properties of these natural compounds are associated with altered expression of several genes or microRNAs that have functional roles in neurodegeneration or cell survival. Our approach could help in quickly assessing multiple natural products for neuroprotective properties and expedite the process of new drug discovery for TBI therapeutics.

## Introduction

Like many thousands of researchers who are engaged in elucidating the underlying mechanisms of and finding therapeutic treatments for traumatic brain injury (TBI) [1–3], we have extensively used animal models of TBI to study the underlying molecular mechanisms [4, 5] and importantly, to test several experimental compounds for potentially neuroprotective effects [6–8]. However, the lesson we have learned from two decades of extensive *in vivo* studies is that evaluating novel compounds for neuroprotective effects in animal models of TBI is a protracted, labor-intensive and costly endeavor with no guarantee of success.

An alternative to animal testing is the use of *in vitro* models of injury [9, 10]. *In vitro* injury models provide a valuable way to rapidly screen potential therapeutic compounds in a relatively moderate throughput manner. Some of the major advantages of drug testing in *in vitro* models is that these studies are easier to implement, considerably less labor intensive, and more cost effective than testing in animal models of brain injury. For our *in vitro* studies, we were informed by our extensive experience studying a region of the medial temporal lobe, the hippocampus, which because of its essential role in learning and memory, is particularly vulnerable to TBI and lifelong disability [11–13]. Rapid stretch injury was initially used as an *in vitro* TBI model by Ellis et al., using primary astrocytes [14] but has since been used with primary hippocampal cells [15] and cerebellar cultures [16]. Since an ideal model of hippocampal injury would use a cell line derived from the hippocampus, we chose for our studies an immortalized hippocampal progenitor cell line, H19-7, that exhibits glial and neuronal lineages characteristic of this brain region [17]. To our knowledge, we are the first to report using this immortalized hippocampal cell line for stretch injury studies with natural product-derived compounds.

Natural products are a primary source of therapeutic drugs, with 60% of drugs on the market derived from natural compounds [18, 19]. The demonstrated neuroprotective properties of some natural products make them potential sources of new therapeutic drugs for neurological disorders [20]. However, one overlooked fact is that these novel compounds are often available in very limited quantities. This precludes testing in animal models of brain injury, but, as we show in our study, their neuroprotective properties can still be investigated in an *in vitro* model of injury.

The three natural compounds we chose to study are clovanemagnolol (CM), vinaxanthone (VX) and pterostilbene (PT). Clovanemagnolol is a compound originally isolated from the bark of the *Magnolina obovata* that can now be synthesized in the lab [21]. This compound has been shown to increase neuronal growth in primary embryonic hippocampal and cortical neurons at doses of 10nM [22] and has previously been shown to increase axonal branching in *Caenorhabditis elegans* by targeting kinesin light chain-1 [23]. Since we were able to obtain sufficient quantities of CM for a small pilot study in our rat model of TBI, we describe our results from both *in vivo* and *in vitro* testing. The second natural product we chose to test is VX which has been previously shown to have remarkable nerve growth promoting effects following injury or transplantation [24, 25]. The third natural product we chose to test, PT, is a dietary compound found naturally in blueberries, which is closely related to resveratrol and has similar antioxidant and anti-inflammatory properties [26]. We reasoned that demonstration of pro-survival and regenerative effects with PT in our *in vitro* system would serve to support the use of this type of testing for rapid screening and discovery of other potentially neuroprotective bioactive compounds in commonly consumed foods as well as provide experimental evidence to suggest dietary changes that could improve brain function in the surviving TBI population.

## Methods

### Pilot study of clovanemagnolol in animals

#### Animals

Adult, male, Charles River Sprague-Dawley rats (300–400 g) were anesthetized with 4% isoflurane, intubated, then mechanically ventilated. A craniotomy was performed laterally to the sagittal suture, midway between the lambda and bregma structures. The fluid percussion device was attached, and the animal was subjected to severe TBI (n = 6) or sham injured (n = 3) as described in Boone et al., [5]. One hour after injury, three of the TBI rats were given 2 mg /kg of CM by IP injection. Rats were sacrificed 24 hours after injury or sham injury and brains were dissected out and placed on dry ice for 10 minutes and stored at -80°C until they were processed.

#### Neuronal counting of injured/dying Fluoro-Jade-positive hippocampal neurons

To test if CM could be neuroprotective in a rodent model of TBI, the number of dying/injured, Fluoro-Jade-positive neurons in the rat hippocampus was determined as previously described in Boone et al., [27]. Briefly, animals were survived for 24 hours, sacrificed and brains dissected out and frozen immediately on dry ice. 10μm coronal rat brain tissue sections were collected on Superfrost Plus slides, every 15^th^ section for 10 sections through the hippocampus. Sections were stained with 0.0001% FJC (a marker for neuronal injury) and neurons were counted in the CA1/2 & CA3 regions using stereological methods [27].

#### Statistical analysis of neuronal counting data

A t-test was performed to compare Severe TBI (n = 3) to Severe TBI+CM (n = 3) within each hippocampal region.

#### Microglial activation

Animals were injected with 2mg/kg of CM 1 hour after injury and survived for 24 hours, sacrificed and brains dissected out and frozen on dry ice. Fresh frozen sections were post-fixed for 20 min in 4°C paraformaldehyde, incubated overnight with 1^o^ antibody (mouse anti-CD11b; 1:2000), the following morning incubated with a 2^o^ antibody (Alexa 594 goat anti-mouse; 1:400) at ambient temperature, and then mounted with nuclear stain DAPI for imaging. Detailed procedure available in Sell et al., [8].

#### Neurogenesis RT^2^ profiler PCR arrays

Neurons from the CA1-CA3 regions of the hippocampus were collected by a PixCell IIe Laser Capture Microdissection Microscope (Life Technologies) from Sham, Severe TBI and Severe TBI + CM-treated rats as previously described in [28]. Cells were lysed in 100μl of lysis buffer and total RNA was isolated using RNAqueous Micro Kit (Ambion). Total RNA was assessed for quality and concentration on an Agilent Bioanalyzer (Agilent Technologies) using the Pico Kit (Agilent Technologies). 10 ng of total RNA was reverse transcribed and pre-amplified using the Qiagen RT2 Pre-amp cDNA synthesis kit along with RT2 PreAmp pathway primer mix specific to genes found in the Neurogenesis Array (Qiagen). QPCR was performed on a Roche Light Cycler 96 using the 96 well format. Data analysis was performed using the ΔΔCT method with Severe TBI and Severe TBI +CM compared relative to sham levels (n = 4/group) [S1 Fig displays genes found in Severe TBI+CM neurons to be significantly differentially expressed compared to Severe TBI alone]. PCR array profiling of laser captured neurons is described in detail in Boone et al., [29]. Bioinformatic and statistical analysis (principal component analysis and hierarchical clustering heatmap analysis) were performed with Qlucore Omics Explorer as previously described in Weisz et al., [30].

#### Statistical analysis of PCR array data

Analysis was performed using R statistical software (R Core Team, 2017, version 3.3.3). In all statistical tests, alpha = 0.05, for a 95% level of confidence. Expression of each gene was modeled by analysis of variance; gene expression as 2^ (-Avg. (Delta (Ct)) were log (base 2) transformed to an approximation of the normal distribution prior to modeling. Differences among treatment groups (TBI, CM) were assessed by Tukey-adjusted contrasts, followed by Benjamini-Hochberg [31] control of the false discovery rate (FDR) among the genes at the 5% level. A table and corresponding graph of genes that were found in the Severe TBI + CM to be statistically different from Severe TBI alone (*p*< 0.05) are shown in S1 Fig.

#### Quantitative real-time PCR analysis of miRNA expression

Neurons from the CA1-CA3 regions of the hippocampus were collected by a PixCell IIe Laser Capture Microdissection Microscope (Life Technologies) from Naive, Severe TBI and Severe TBI + CM treated rats. Cells were lysed in 100μl of lysis buffer and total RNA was isolated using RNAqueous Micro Kit (Ambion). Total RNA was assessed for quality and concentration on an Agilent Bioanalyzer (Agilent Technologies) using the Pico Kit (Agilent Technologies). 1 ng of total RNA was reverse transcribed using the Taqman MicroRNA kit (Applied Biosystems). QPCR was performed on a Roche Light Cycler 96 using the microRNA Taqman probes to miR-212 and miR-9 (n = 3 biological replicates). Data analysis was performed using the ΔΔCT method with Severe TBI and Severe TBI +CM compared relative to naive levels.

### *In vitro* studies

#### Culture of H19-7 cells

Collagen I coated 6-well BioFlex plates (Flexcell International, Burlington, NC) were coated overnight with 0.01% poly-L-lysine (Sigma-Aldrich). The solution was removed and wells were rinsed three times with sterile water and allowed to dry before use. H19-7 undifferentiated fibroblasts (ATCC, CRL-2526) were plated at a density of 70,000 cells per well in growth media consisting of high glucose DMEM (Gibco), 10% Fetal Bovine Serum and 1.0% Pen-Strep. H19-7 cells were incubated at 34⁰C in 10% CO_2_ for 48 hours. After 48 hours media was changed to high glucose DMEM (Gibco) with 1% N2 supplement and bFGF. Cells were given 24 hours to differentiate before drug treatment.

#### Drug treatment of cells

Please note that given the limited quantities of some of the experimental natural products that were available for *in vitro* testing, the lowest possible drug doses were chosen empirically based on previous reports in the literature combined with a series of preliminary experiments testing drug doses from 1nM to 100μM. For the data shown in this report, clovanemagnolol (CM) was added to a final concentration of 10nM to each well 48 hours prior to stretch injury and the cell viability (MTS) assay. Media and drug were replaced at 24 hr prior to injury. Vinaxanthone (VX) was added to a final concentration of 8.675 μM 48 hr prior to stretch injury and the drug and media replaced daily. Pterostilbene (PT) was added to a final concentration of 1μM 48 hrs before stretch injury. Two other natural compounds, a dietary anthocyanin found in blackberries, cyanidin-3-O-β-glucopyranoside (aka cyanidin-3-O-β-glucoside) and amphotericin B, a polyene antifungal antibiotic, were tested but had no significant effect on cell growth or stretch injury (data, including drug doses, are shown in S2 Fig. Small quantities of clovanemagnolol and vinaxanthone were provided by Dr. Siegel and the biosynthesis of these compounds are described in detail in Cheng et al. [21] and Axelrod et al, [24]. Pterostilbene and cyanidin-3-O-glucoside (S1 Data) were purchased from Cayman Chemical (Ann Arbor, Michigan) and amphotericin B (S1 Data) obtained from Sigma-Aldrich (St. Louis, MO).

#### *In vitro* stretch injury

**Fig 1:**
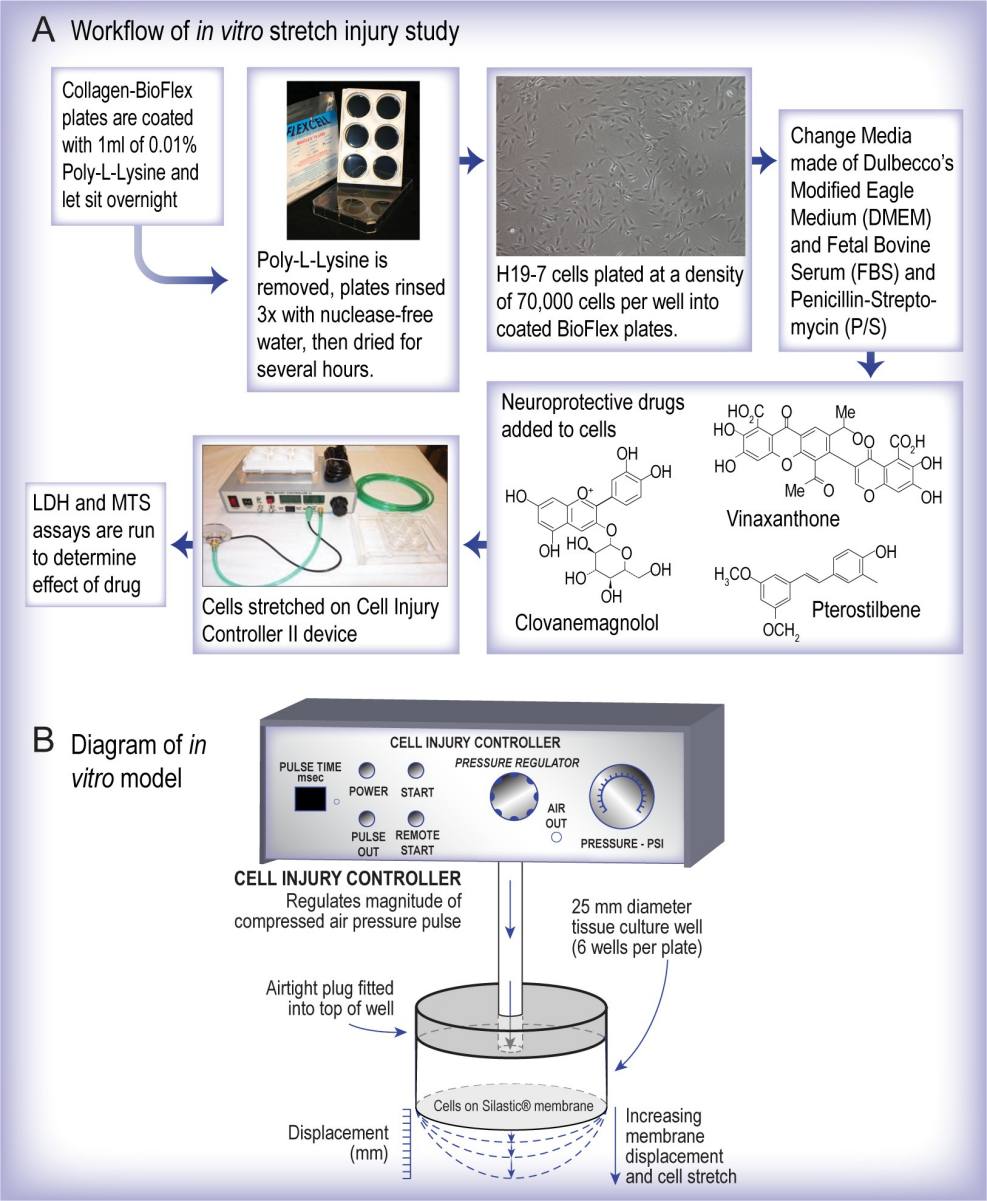
Research design and *in vitro* model. **(A)** Workflow of the *in vitro* stretch injury study. **(B)** Diagram illustrating the mechanism by which the 50 msec, 50 psi stretch injury is delivered onto the cells. The airtight plug is fitted into the well and delivers the burst of nitrogen gas. Figure adapted from Cohen et al. Prog Brain Res. (2007).161:143–69.

The workflow of the *in vitro* stretch injury experiments is shown in Fig 1A. The in vitro stretch injury system is shown in Fig 1B. Differentiated H19-7 cells in Bioflex plates were stretched using the Cell Injury Controller II (Custom Design and Fabrication, Richmond, VA). The device uses nitrogen gas to produce a controlled pressure pulse which deforms the membrane of the flex plates well producing an injury. Following preliminary studies, two levels of injury were examined in these studies; moderate injury with a 3.9 peak injury pressure (50 msec duration, 30.0 psi regulator pressure) and a severe injury with a 6.3 peak injury pressure (50 msec duration, 50.0 psi regulator pressure). Please note that, except for the CM study, only severe injury data are presented here for all other drugs. All stretch injury experiments with natural products were independently repeated four to six times.

#### MicroRNA-seq and bioinformatic analysis of vinaxanthone and pterostilbene data

Lysis buffer from the RNAqueous Micro kit was used to collect H19-7 cells (control, drug-treated) which were rinsed with RNase-free water before cell lysis. Total RNA was made according to manufacturer’s protocols and microRNA sequencing analysis was performed at the UTMB sequencing core facility and subsequent bioinformatic analysis was performed with Qlucore Omics Explorer as described in Weisz et al., [30].

#### Propidium iodide staining

Cell Injury level was assessed using propidium iodide (PrI), a marker of compromised cell membranes (Life technologies) and Hoechst 3342 (Life technologies). 10 μL of 1mg/mL was added to the 2 mL of media in each well to reach a concentration of 5μg/mL and incubated in a 39⁰C incubator with 10% CO_2_ for 30 minutes. Hoechst 3342, 1.5 μL of a 10mg/mL solution, was added to 2 mL of media in each well to reach a concentration of 7.5 μg/mL and incubated at 39⁰C with 10% CO_2_ for 10 minutes. Dyes were replaced with 2 mL of fresh media before imaging. PrI and Hoechst 3342 dyes were imaged using a 559 nm and 405 nm lasers, respectively. Five images under 20x were taken of each well starting blindly in the center of the well, and taking images to both sides, above, and below. PrI positive cells and Hoechst 3342 cells were counted manually using the 16-bit images in Image J software (NIH). Cell Injury was expressed as the percentage of injured cells. The 50 msec 30 psi moderate injury and 50 msec 50 psi severe injury level corresponded to a 25% and 57% cell injury, respectively. Cell injury assessments were performed independently three times.

#### Immunohistochemistry for MAP2

Coverslips in 24 well plates were coated with 0.01% poly-L-lysine and H19-7 cells were plated 40,000 cells/well in DMEM growth media (10% FBS and 1% Penstrep). 24 hours later media was changed to DMEM media containing N2 supplement and bFGF. Cells were fixed using 4% paraformaldehyde, rinsed in 1X PBS and blocked in 5% normal goat serum/0.3% Trition X-100 in PBS. Cells were incubated in a primary antibody Anti-Map2 (1:200, Millipore # MAB3418) in 1.5% normal goat serum/0.3% Trition X-100 in PBS overnight at 4⁰C. Cells were incubated in a goat anti-mouse ALEXA- 488 (1:400) conjugated antibody diluted in 1.5% normal goat serum/0.3% Trition X-100 at RT for 1 hour. Cells were rinsed with PBS, dH20 and stained with DAPI for 5 minutes. Cells were rinsed in ddH20 and mounted with FluorSave (Calbiochem). Slides were viewed on an Olympus BX51 microscope under FITC and DAPI filters. Images were captured at 10x and 20x using Picture Frame software.

### Cell viability (MTS) and cell toxicity (LDH) assays

#### LDH assay

Media samples were taken from each well of flex plate before stretch, 4hr and 24hr following injury. Samples were tested for LDH levels [32] using the Pierce LDH Cytotoxicity Assay Kit (Thermo-Fisher). Results were read using a Promega Glomax Multi Detection System Plate Reader using 490 nm and 690 nm filters.

#### MTS assay

Cell viability was assessed using the CellTiter 96 Aqueous Non-Radioactive Cell Proliferation Assay (Promega). 100μL of the MTS reagent was added to each 1 mL of media in flex plates and were returned to the incubator for 100 minutes. Results were read using 100μL of solution in a 96 well plate using a Promega Glomax Multi Detection System Plate Reader using 490 nm and 690 nm filters.

### Statistical analysis

Statistical analyses were conducted in R Studio, utilizing a two-way ANOVA with post Tukey multiple comparisons of the means.

Data availability: All relevant data files are provided in S1 and S2 Figs and S1 Data.

## Results and discussion

The *in vitro* and *in vivo* studies described herein were performed in parallel because we quickly realized that we only had sufficient quantities of CM to test in a few rats as a pilot study. We lacked sufficient quantities of the other natural product-derived compounds for *in vivo* testing so all other compounds were only tested *in vitro*.

**Fig 2:**
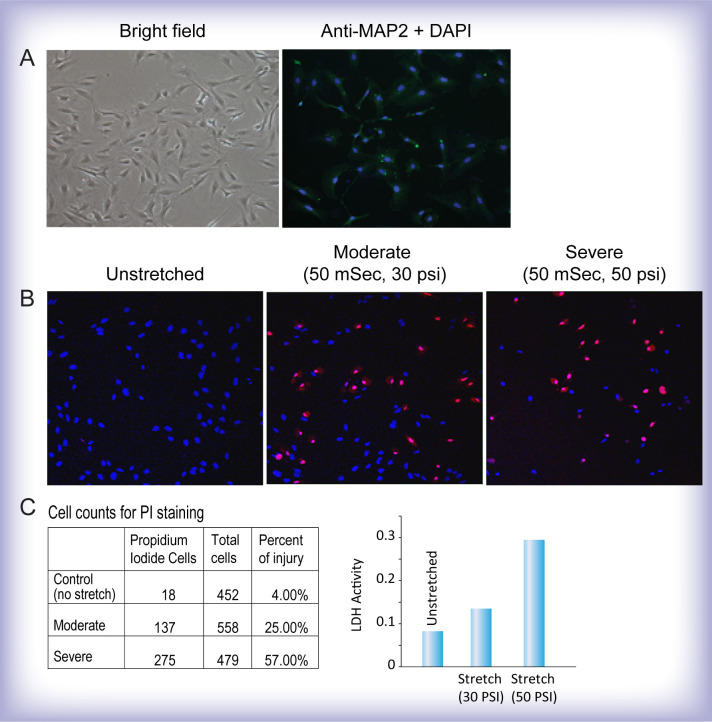
Characterization of the immortalized H19-7 hippocampal cell line. **(A)** H19-7 cells shown in bright field and stained positive with a neuronal marker Anti-MAP2 and DAPI. **(B)** Stretch injury levels were assessed using propidium iodide (PrI, red) and Hoechst 3342 (blue) staining. Unstretched H19-7 hippocampal neurons showed few PrI-stained cells. Increasing numbers of PrI-positive cells were detected in H19-7 cells subjected to a moderate injury level of 50 msec, 30 psi of pressure (~25% cell injury) and a more severe injury level of 50 msec, 50 psi (~57% cell injury). **(C)** The cell counts for PrI staining corresponded to increased levels of lactate dehydrogenase (LDH), a marker of tissue damage, released from stretch-injured cells.

For the *in vitro* stretch injury studies, we first determined that H19-7 cells stained positive with an antibody to the neuronal marker, microtubule associated protein 2 (MAP2), a marker expressed only in neuronal cell types, to confirm the use of these cells as a neural cell type (Fig 2A). Next, we determined that moderate and severe rapid stretch injury increased PrI staining, 25% and 57%, respectively, of all stretched cells (Fig 2B and 2C); the increased levels of cellular damage were also confirmed with LDH assays of the supernatant of the stretched cells (A representative dataset from one experiment is shown in Fig 2C).

**Fig 3:**
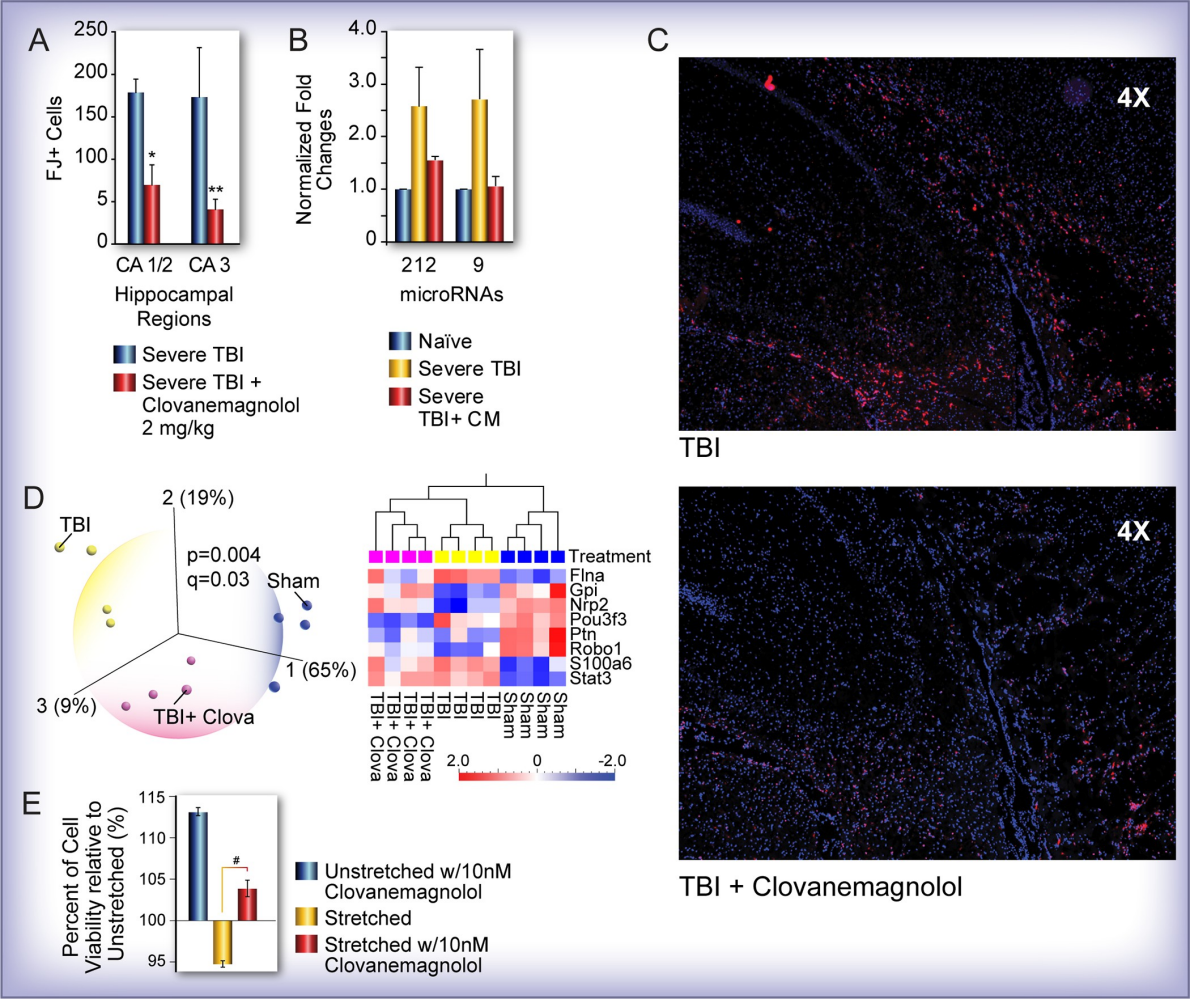
Characterization of the neuroprotective effects of clovanemagnolol (CM) *in vivo* and *in vitro*. **(A)** Stereological counting of dying/injured Fluoro-Jade-positive (FJ+) pyramidal hippocampal neurons in TBI and TBI+CM rats showed that CM treatment significantly reduced neuronal injury in the CA1/2 (*p<0.02) and CA3 (**p<0.04) regions. **(B)** Quantitative real-time RT-PCR analysis of microRNA expression in the CA1-CA3 hippocampal subregions showed that CM treatment appeared to restore expression of miR-212 and miR-9 to naïve control levels. Results did not reach statistical significance. **(C)** Immunofluorescent staining of relative microglial activation (anti-CD11b) between TBI and TBI + CM-treated rats. The images show the hippocampal formation and surrounding regions. **(D)** Gene expression data from the RT profiler Neurogenesis PCR arrays (n = 4/group) were analyzed using bioinformatic software (Qlucore Omics Explorer). Principal component and hierarchical clustering heatmap analysis of significantly expressed genes (p = 0.004, q = 0.03) in the hippocampus of sham control, TBI and TBI+CM rats shows that eight genes can clearly discriminate the three groups from each other. Two genes (Gpi and Nrp2) that are restored to sham control levels by CM are associated with pro-survival roles in the brain. **(E)** H19-7 cells treated with 10nM CM 48 hours pre-injury were stretched at 50 msec, 30psi. A significant increase in cell viability of the drug treated stretched cells compared with stretched cells was shown by an MTS assay 24 hours after injury (#p<0.05). All error bars in A, B and E are SEM, standard error of the mean.

Cell counts from an *in vivo* pilot study of CM effects 24 hours post-TBI show that CM (2mg/kg) was significantly neuroprotective; CM treatment of TBI rats led to a significant reduction in the numbers of Fluoro-Jade-positive cells, a marker of dying neurons, in both the CA1/2 (p<0.02) and CA3 (p<0.04) regions of the CM-treated hippocampus compared to TBI alone (Fig 3A). To gain insight into the underlying neuroprotective mechanisms and given our longstanding interest in the role of microRNAs (miRNAs) in the pathogenesis of TBI [5], we performed real-time quantitative PCR analysis of laser captured CA1-CA3 hippocampal neurons using Taqman probes for two miRNAs (miR-212 and miR-9) implicated in TBI pathology. CM treatment appeared to restore TBI-induced increased expression of miR-212 and miR-9 to naïve control levels, albeit not quite reaching statistical significance (Fig 3B). To assess the effects of CM treatment on neuroinflammation, we performed immunohistochemical analysis of hippocampal sections using CD11b, a marker of microglial activation that serves as an effective readout of TBI-induced inflammation and found that CM treatment reduced microglial activation in the hippocampal formation (Fig 3C).

Next, to gain further mechanistic insight into the neuroprotective effects of CM, we performed pathway-specific PCR array (Neurogenesis array) analysis of laser captured hippocampal neurons from sham injured, TBI and TBI plus CM rats. We performed principal component and hierarchical clustering heatmap analysis of significantly altered genes (Fig 3D) and found that a small number of genes previously implicated in cell survival and hippocampal function were restored to uninjured control levels by CM (S1A and S1B Fig). Notably, two genes (Gpi and Nrp2) whose expression levels were decreased by TBI in the rat hippocampus, were upregulated back to sham levels. Gpi (aka neuroleukin) which is known to be involved in brain development, was previously found expressed in reactive astrocytes and implicated in axonal regeneration in the injured mouse brain [33]. Gpi protein expression was previously shown to be decreased in diabetic rats subjected to experimental ischemic brain injury [34]. Mice deficient in the second gene, Nrp2, that is also upregulated and restored to sham control levels by CM, have been shown to have impaired hippocampal-dependent memory and motor function [35]. In our *in vitro* studies of CM on control and stretched H19-7 cells, we used an MTS cell viability assay to show that a significant decrease in cell viability can be measured 24 hours after stretch injury, and when stretched cells were treated with 10nM dose of CM, cell viability levels were significantly increased compared to stretch alone (p<0.05) (Fig 3E).

**Fig 4:**
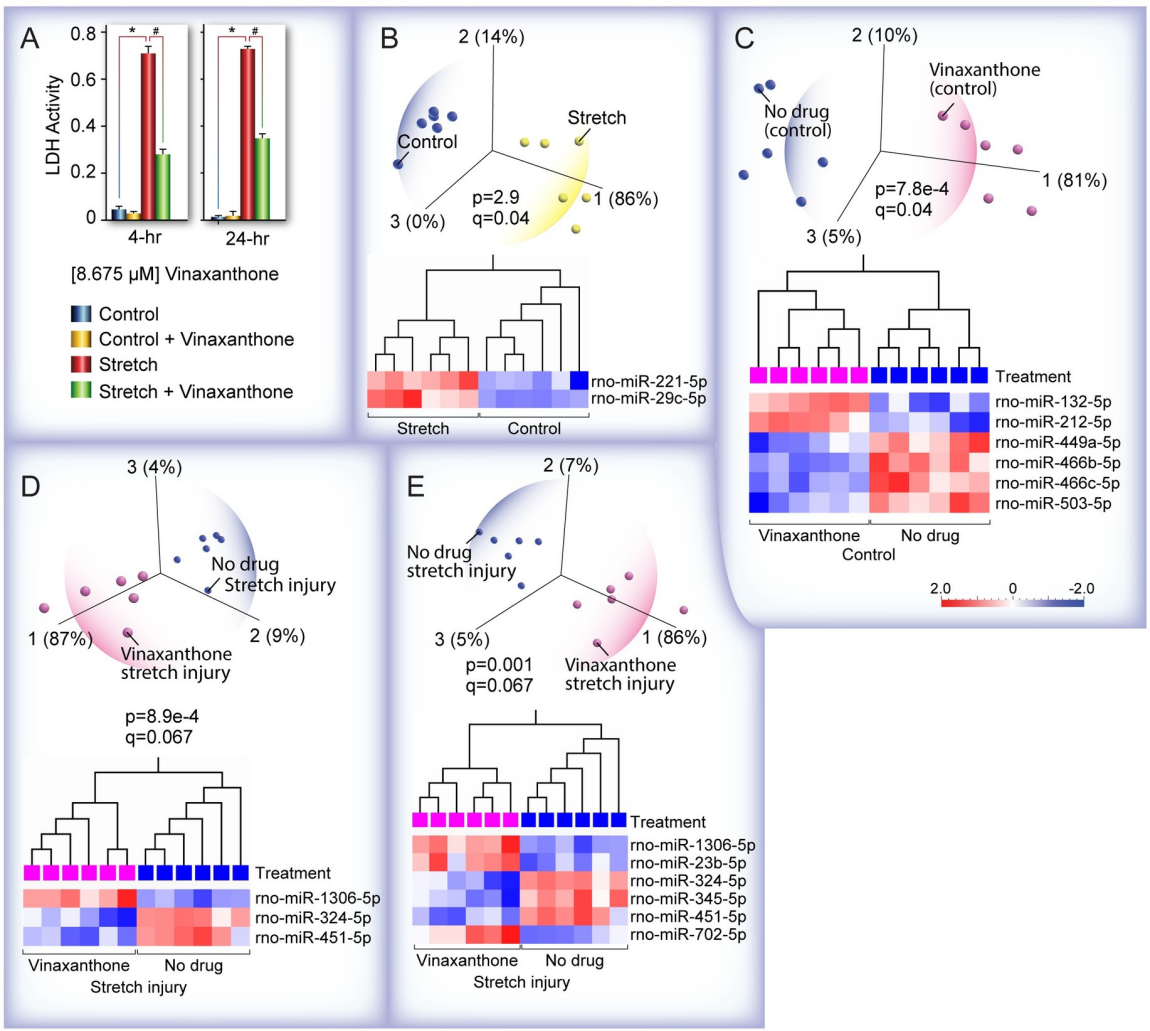
Characterization of the pro-survival effects of vinaxanthone (VX) *in vitro*. **(A)** H19 cells pre-treated with 8.675 uM of vinaxanthone 48 hrs prior to stretch injury were stretched (50 msec, 50 psi) and 4 and 24 hours later, media from each well was collected for LDH assays (n = 6). LDH release was elevated in stretch wells compared to control (*p < 0.001). Stretched + drug cells released less LDH compared to stretch alone wells (#p < 0.001). Error bars = SEM, standard error of the mean **(B)** Principal component analysis (PCA) and hierarchical clustering heatmap of microRNA (miRNA) expression in H19-7 cells showing that only two differentially expressed miRNAs distinguish stretch-injured from uninjured control cells. **(C)** PCA and hierarchical clustering heatmap of miRNA expression in VX-treated control and untreated H19-7 cells show that differential expression of only six miRNAs, whose target genes are known to be essential for critical brain functions and neuronal survival, clearly identify the VX-treated from untreated control cells. **(D)** PCA and hierarchical clustering heatmap of miRNA expression in stretch injured neurons showed that VX alters expression of miRNAs implicated in modulation of hippocampal spatial memory (miR-324-5p) and proliferation and migration of Schwann cells (miR-451-5p). **(E)** PCA and hierarchical clustering heatmap analysis of miRNA expression at a lower stringency p value cutoff showed that VX upregulated miR-702-5p which is known to inhibit apoptosis-related genes.

The reported regenerative properties of VX suggested that this natural compound has neuroprotective effects which could be studied in stretch injured hippocampal neurons. We first examined the neuroprotective effects of VX on stretch injured neurons using the LDH assay. VX had no observable effect on control cells but significantly ameliorated cell damage (p<0.001) at both 4 and 24 hours post-injury (Fig 4A). To further examine potential molecular mechanisms of drug effects, we collected the cells from the VX stretch injury experiments, made total RNA and subjected the samples to microRNA sequencing (miRNA-seq) analysis. To determine the effects of stretch injury on miRNA expression levels in the absence of drug treatments, we first compared miRNA expression in control and stretched H19-7 cells and using PCA and hierarchical clustering heatmap analysis, we found that only two differentially expressed miRNAs (miR-221-5p and miR-29c-5p) could distinguish stretch-injured from uninjured control cells (Fig 4B).

Next, we compared miRNA expression in control cells with and without VX treatment (Fig 4C). We show that differential expression of only six miRNAs clearly identify the VX-treated cells. Notably, miR-132-5p and miR-212-5p, which are significantly increased by VX treatment in control cells, are found downregulated in post-mortem brains of Alzheimer’s disease patients [36]. MiR-132 is also found downregulated in CSF and serum of sporadic ALS patients [37], suggesting that the miRNA gene targets (which would be predicted to be upregulated if the miRNAs that regulate them are downregulated) are associated with neurodegenerative pathology. A clue to one of the neuroprotective mechanisms is that increased levels of miR-212-5p has been shown to be protective after TBI [38] and shown to inhibit neuroinflammation [39].

An additional insight into the protective effects of VX treatment is that one of the predicted gene targets of miR449a-5p (downregulated in H19 cells by VX treatment) is synaptotagmin 1 (*Syt1*), a calcium sensor involved in exocytosis that is essential for synaptic plasticity [40] and is predicted to be upregulated by VX. One way to understand the biological relevance of miRNAs is to examine what is known about the functional roles of their predicted target genes using miRNA prediction algorithms such as Targetscan; analysis of predicted gene targets of miRNAs downregulated by VX in control cells (miR-449a-5p, miR-466b/c-5p, miR-503-5p) revealed that many of these miRNA target genes are known to be essential for critical brain functions and neuronal survival [41].

We gained further key insights into the underlying mechanisms of VX-mediated neuroprotection by examining the known biological functions of two miRNAs that are downregulated by VX in stretch injured neurons (Fig 4D); downregulation of miR-324-5p is linked to modulation of spatial memory in the hippocampus [42] and notably, syringic acid, a natural product with neuroprotective activity *in vivo* has been shown to promote the proliferation and migration of Schwann cells via the downregulation of miR-451-5p [43]. We found another clue to the pro-survival mechanisms induced by VX in a rat study of miRNAs associated with the pathogenesis of impaired fracture healing caused by diabetes mellitus (DM); miR-451-5p was significantly upregulated in DM rats with femoral shaft fractures [44]. Interestingly, using a lower stringency p value cutoff (Fig 4E), we found that miR-702-5p which is upregulated by VX treatment in stretch-injured cells, is implicated in inhibiting apoptosis-related genes [45]. A final clue to the underlying pro-survival mechanisms of VX is that miR-1306-5p, most significantly upregulated (p = 2.6e-5, q = 0.067) by VX, has been shown to protect against ischemia/reperfusion injury *in vitro* by targeting and inhibiting a pro-apoptotic gene, Bik (Bcl2-interacting killer) [46].

**Fig 5:**
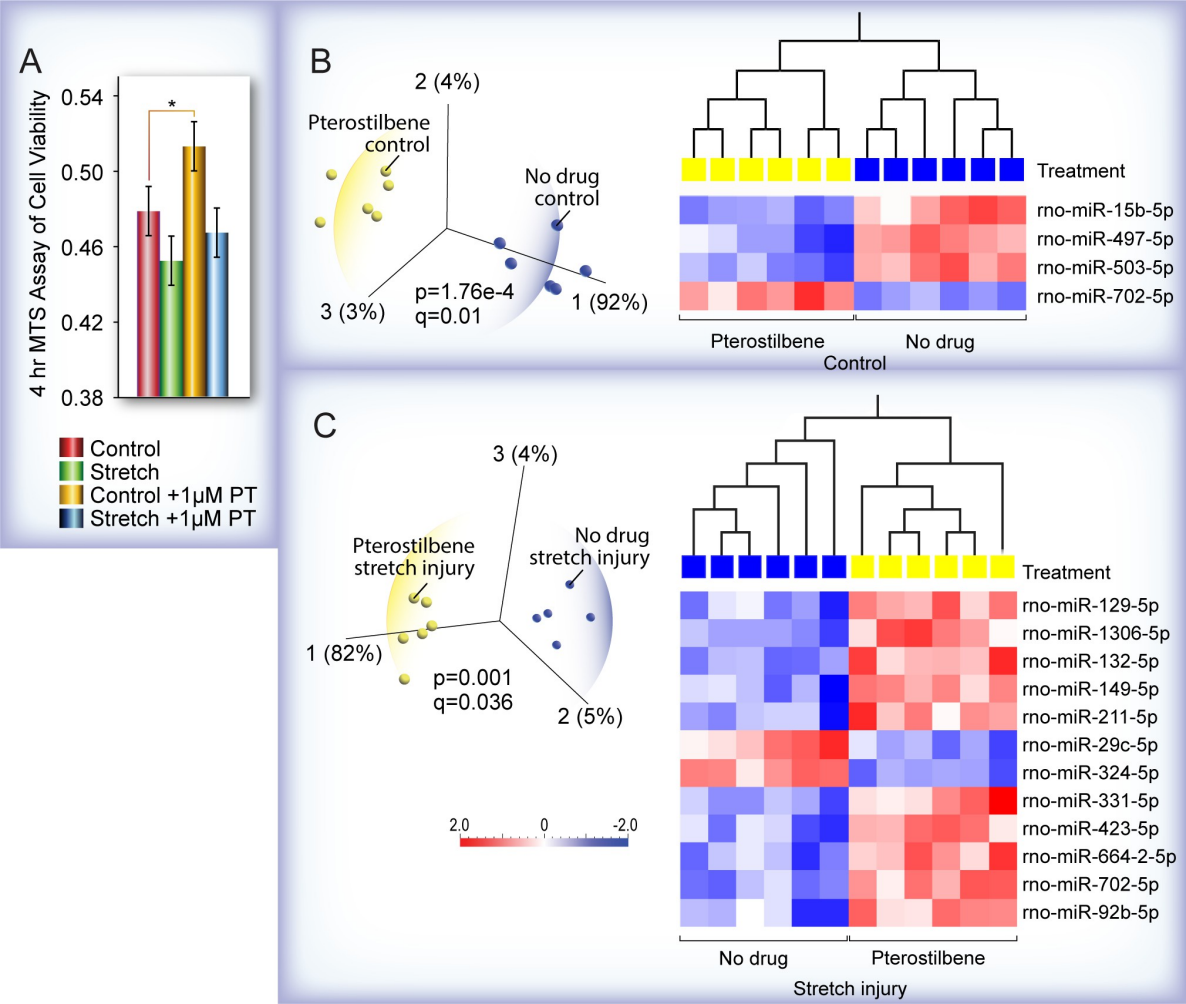
Characterization of the pro-survival effects of pterostilbene (PT) *in vitro*. **(A)** PT significantly increased cell viability of unstretched control H19-7 cells (*p<0.02). Error bar = SEM, standard error of the mean **(B)** PCA and hierarchical clustering heatmap analysis of miRNA expression in control cells showed that PT treatment downregulated three miRNAs which are known to target the pro-survival genes Bdnf and Bcl-2. **(C)** Although PT treatment had no apparent effects on the viability of stretch injured H19-7 cells as measured by the MTS assay, PCA and hierarchical clustering heatmap analysis revealed that PT downregulated two miRNAs (miR-29c-5p, miR-324-5p) whose altered expression could serve as a molecular marker of PT-induced pro-survival effects.

We next analyzed the effects of PT in H19-7 cells. Since the LDH assay did not show statistically significant effects of PT on stretch injury, we used the MTS assay to measure cell viability in control and stretched cells treated with PT. Although again, we did not find any significant effects on stretch-injured cells, PT treatment did significantly increase cell viability (Fig 5A). PCA and hierarchical clustering heatmap analysis of miRNA expression in control cells (Fig 5B) showed that PT treatment downregulated three miRNAs (miR-15b-5p, miR-497-5p, and miR-503-5p) which are known to target the pro-survival genes Bdnf and Bcl-2; because an individual miRNA and its target gene expression is inversely correlated, this suggests that Bdnf and Bcl-2 are upregulated. This result is concordant with the pro-survival effect of PT on the control cells. Notably, as we found with VX, PT treatment also significantly upregulated miR-702-5p, which is implicated in inhibiting apoptosis-related genes [45].

One important point related to the PT experiments is that a single assay should not rule out a potential drug candidate and sometimes a molecular analysis can shed mechanistic insights into the efficacy of a therapeutic compound. Although we did not find significant effects of PT on stretch-injured neurons, PCA and hierarchical clustering heatmap analysis of the miRNA-seq data from the stretch-injured cells helped us uncover possible mechanisms for the pro-survival effects of PT (Fig 5C). Like VX, PT treatment downregulated expression of miR-324-5p whose decreased expression is associated with improvement in hippocampal spatial memory. MiR-29c-5p was also recently identified as involved in the neuropathology of and potential biomarker of AD, depression, schizophrenia and ionizing radiation-induced brain damage [47]. Thus, the suppression of miR-29c-5p expression in PT-treated stretch-injured H19-7 neurons suggests that miR-29c-5p as well as miR-324-5p could serve as markers of PT-induced pro-survival effects. Targetscan analysis also revealed that many of the gene targets of miR-29c-5p are associated with pro-survival functions.

The consensus in the literature is that PT is neuroprotective and several mechanisms have been proposed including inhibition of microglial activation [48], and anti-inflammatory oxidative stress-induced signaling [49]. Our study shows that PT treatment significantly increased cell viability in control cells and the underlying mechanism involves suppression of miRNAs that target two pro-survival genes, Bdnf [50] and Bcl-2 [51] and upregulation of an anti-apoptotic miRNA, miR-702-5p [45]. Further validation of our results come from a study by Rege et al., who used H19-7 hippocampal cells to show that resveratrol, closely related to PT, protects from beta-amyloid-induced oxidative damage [52].

In conclusion, we have used *in vitro* screening methods to show that the neuroprotective properties of natural compounds can be rapidly evaluated. We were able to perform a pilot study in a rat model of TBI with CM and confirm its neuroprotective effects using our *in vitro* assays. We have also used PCR array and miRNA-seq analysis of the cell and tissue samples from these assays to show that the neuroprotective effects of CM, VX and PT are associated with altered expression of several genes or microRNAs that have functional roles in neurodegeneration or cell survival. This approach could help expedite the screening of other natural product compounds for TBI therapeutics.

## Supporting information

S1 Fig Neurogenesis PCR array. (TIF)

S2 Fig Stretch injury studies with C3G and amphotericin B. (TIF)

S1 Data (XLSX)
