# Supplementary figures and images for: Mechanistic insights gained from cell and molecular analysis of the neuroprotective potential of bioactive natural compounds in an immortalized hippocampal cell line
Source: PLoS One. 2022 Jun 3;17(6):e0267682. doi: 10.1371/journal.pone.0267682 (PMC9165808; doi:10.1371/journal.pone.0267682)

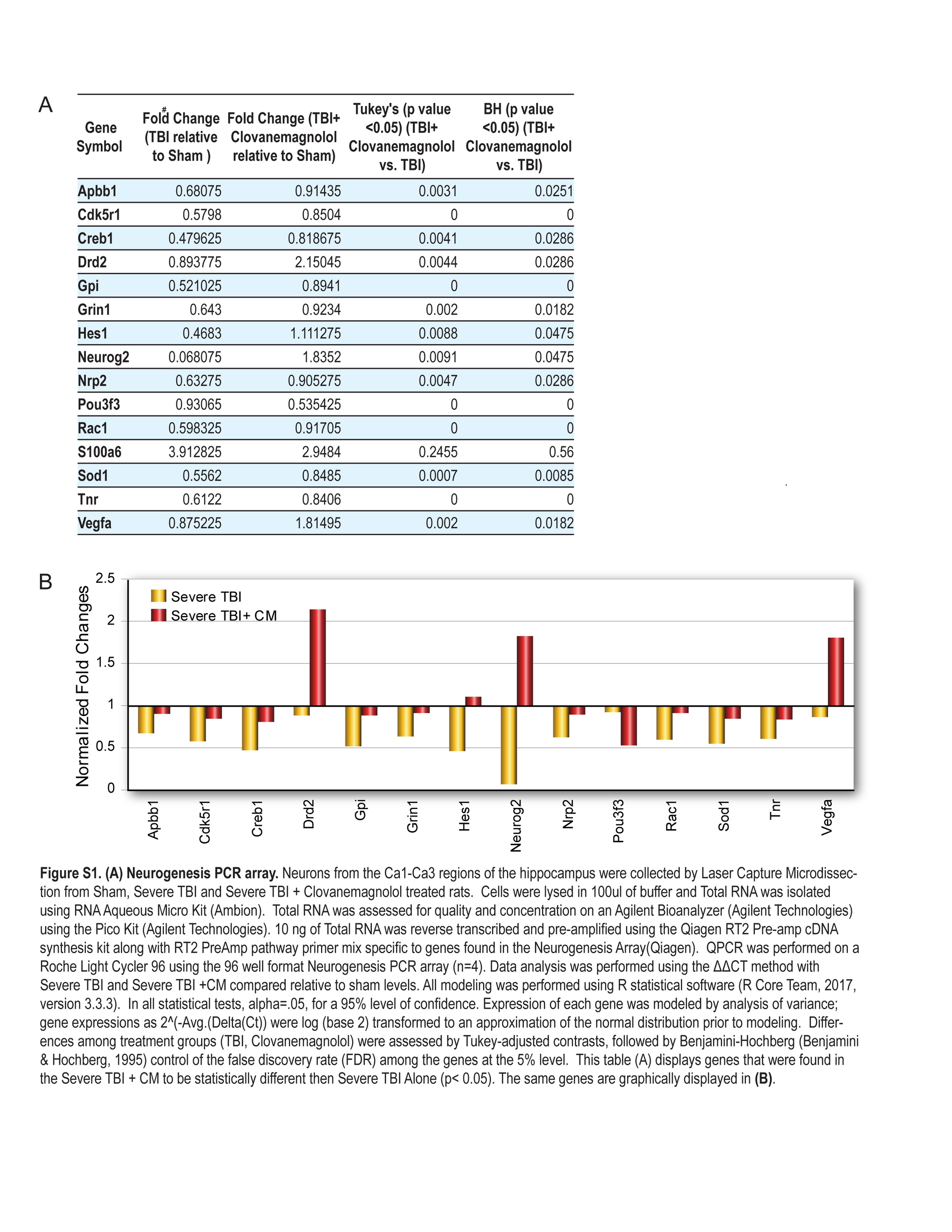

Supplement: S1 Fig — (TIF) [file pone.0267682.s001.tif]

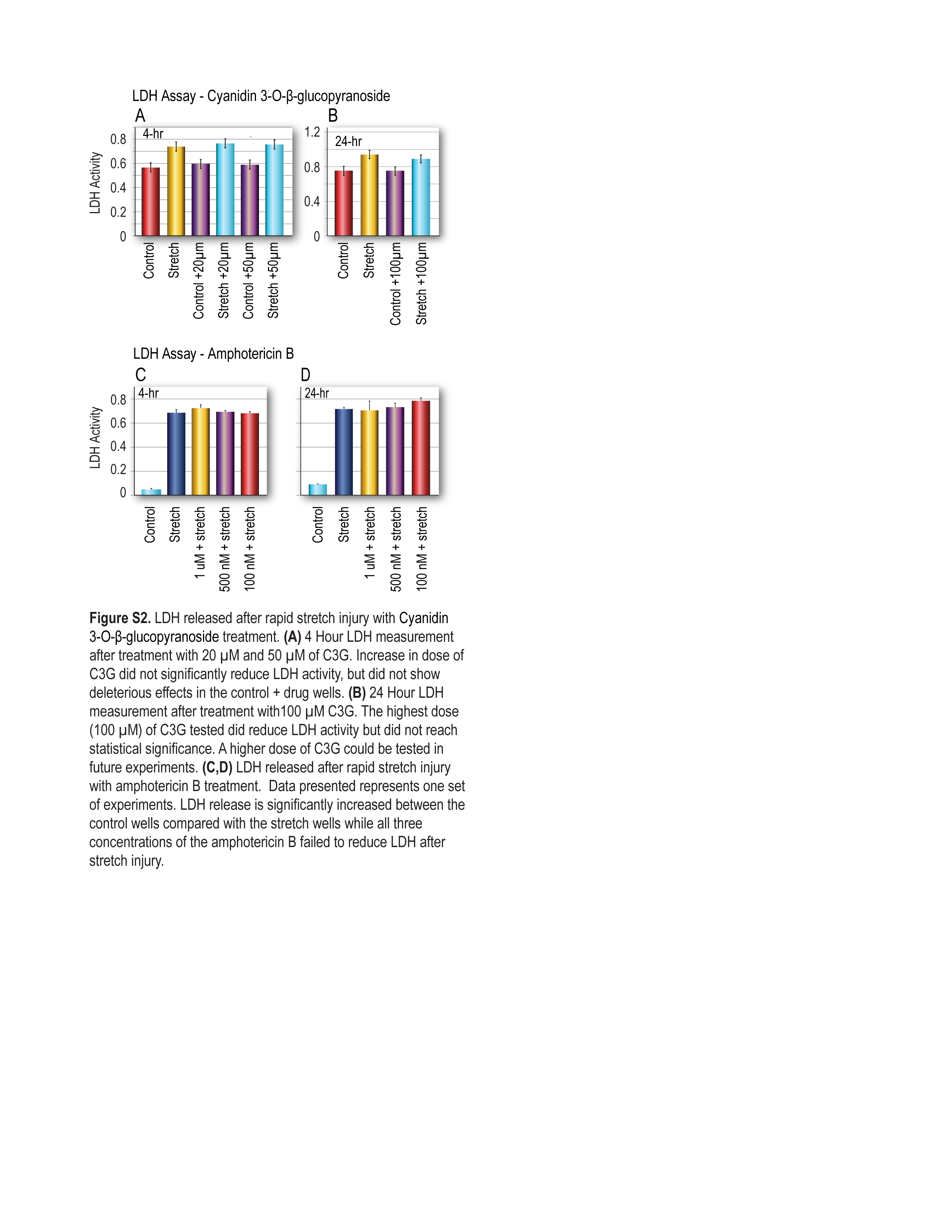

Supplement: S2 Fig — (TIF) [file pone.0267682.s002.tif]
